# Sulfur Amino Acid Metabolism and the Role of Endogenous Cystathionine-γ-lyase/H_2_S in Holstein Cows with Clinical Mastitis

**DOI:** 10.3390/ani12111451

**Published:** 2022-06-04

**Authors:** Bohao Zhang, Ting Lin, Xu Bai, Xiaoxiao An, Lijun Dai, Jun Shi, Yong Zhang, Xingxu Zhao, Quanwei Zhang

**Affiliations:** 1 College of Veterinary Medicine, Gansu Agricultural University, Lanzhou 730070, China; zhangbhgs@163.com (B.Z.); zhangy@gsau.edu.cn (Y.Z.); 2 College of Life Science and Biotechnology, Gansu Agricultural University, Lanzhou 730070, China; lint0811@163.com (T.L.); b1820313351@126.com (X.B.); a1732036832@163.com (X.A.); d1312496231@163.com (L.D.); sj18034650439@163.com (J.S.); 3 Gansu Key Laboratory of Animal Generational Physiology and Reproductive Regulation, Lanzhou 730070, China

**Keywords:** clinical mastitis, hydrogen sulfide, cystathionine-γ-lyase, inflammation

## Abstract

**Simple Summary:**

Endogenous hydrogen sulfide (H_2_S) has been implicated in many physiological and pathological processes, particularly in inflammatory responses and adaptive immunity. In this study, we identified the candidate differentially expressed proteins (DEPs) associated with H_2_S metabolism in Holstein cows with clinical mastitis (CM). The results revealed 17 DEPs included in 44 Gene Ontology (GO) terms and five Kyoto Encyclopedia of Genes and Genomes (KEGG) pathways related to sulfide metabolism and indicated the important role of cystathionine-γ-lyase (CTH)/H_2_S in CM. Our findings can support research into the function and regulatory mechanism of CTH/H_2_S in Holstein cows and provide a basis for the prevention and treatment of CM.

**Abstract:**

H_2_S plays an important role in various inflammatory diseases. However, the role of H_2_S and synthetic enzymes in Holstein cows with CM is unknown. The aim of this study was to identify DEPs associated with sulfide metabolism and further investigate their roles in dairy cows with CM. From 3739 DEPs generated by data-independent acquisition proteomics, we identified a total of 17 DEPs included in 44 GO terms and five KEGG pathways related to sulfide metabolism, including CTH and cystathionine-β-synthase (CBS). Immunohistochemical and immunofluorescence staining results showed that CTH and CBS proteins were present mainly in the cytoplasm of mammary epithelial cells. Endogenous H_2_S production in the serum of the CM group was significantly lower than that of the healthy Holstein cows. *CTH* and *CBS* mRNA and protein levels in the mammary glands of the CM group were significantly downregulated compared to those of the healthy group. These results indicate that CTH and H_2_S were correlated with the occurrence and development of CM in Holstein cows, which provides important insights into the function and regulatory mechanism of CTH/H_2_S in Holstein cows.

## 1. Introduction

Clinical mastitis (CM) in cows describes an inflammatory response of the mammary glands stimulated by a variety of factors [1], which leads to huge economic losses in the global dairy industry. CM is easily detectable in dairy cows through visible abnormalities [1,2]; however, the control and prevention of CM are hindered by its complex etiology and multifactorial nature [3,4]. Antimicrobial agents are frequently used to prevent and treat CM in mammals. Approximately 24% of antibiotics in the dairy industry are used for mastitis treatment and approximately 44% are used for mastitis prevention [5]. However, the generation of drug residues and the emergence of antibiotic-resistant pathogens have increased the difficulty and cost of CM treatment in mammals and pose continuous risks to human health and food safety [6,7]. Thus, it is becoming increasingly urgent to develop novel prevention and treatment methods and non-antibiotic-resistant medicines [8]. Previous studies have investigated vaccine development, searched for new therapeutic alternatives to antibiotics, and even explored the application of traditional Chinese medicine to veterinary medicine [9]. However, further advances in CM treatment in mammals are still required. Thus, additional studies, drugs, and methods are required to improve prophylactic and therapeutic approaches for CM from different perspectives, for example, considering their anti-inflammatory effects, enhanced immunity, and accelerated fluid circulation.

Endogenous hydrogen sulfide (H_2_S), a gaseous transmitter, is in relative equilibrium in animal organisms and plays an important physiological regulation function. Abnormal changes in its concentration can lead to a variety of diseases related to the digestive system, cardiovascular system, nervous system, urinary system, and so forth [10]. Coincidentally, H_2_S has been implicated in many physiological and pathological processes, particularly inflammatory responses and adaptive immunity [10,11]. Research shows that H_2_S exerts anti-inflammatory effects by reducing the secretion of inflammatory cytokines and increasing the levels of anti-inflammatory and cytoprotective molecules [11]. H_2_S can modulate the activities of several immune cells, including monocyte and polymorphonuclear cell apoptosis, leukocyte adhesion and infiltration, T cell activation, proliferation, and inflammatory cytokine production. However, many studies have indicated that H_2_S may play a dual role in the inflammatory process [10]. For example, in the model of septic shock caused by cecal ligation and perforation, H_2_S can promote leukocyte proliferation and then aggravate the pulmonary inflammatory response [12]. On the other hand, H_2_S can not only significantly inhibit aortic atherosclerotic plaque, but also inhibit neutrophil aggregation in lung tissue, reduce pulmonary edema, and increase pulmonary capillary permeability [13]. However, the roles of H_2_S and its synthetic enzymes in cow CM are not completely understood. Endogenous H_2_S is synthesized by specific enzymes including cystathionine-β-synthase (CBS), cystathionine-γ-lyase (CSE/CTH), 3-mercaptopyruvate sulfurtransferase (3-MST), and cysteine aminotransferase (CAT) [14,15]. These enzymes are extensively expressed in mammalian tissues, such as the liver, kidney, and spleen, and are induced by external stimulations and intracellular environments [16,17]. However, it is unclear whether these enzymes are expressed in mammary glands, particularly in mammary glands with CM. H_2_S exerts its biological effects by regulating the activity of kinases, phosphatases, transcription factors, and ion channels via a variety of signaling pathways [18]. Many of these actions are attributed to a post-translational modification of L-cysteine (Cys) residues, which is referred to as sulfhydration or persulfidation [19]. Cys is a non-essential α-amino acid with disulfide bonds and a nonpolar sulfhydryl (-SH) group that is abundantly present in milk, which also provides more substrates for endogenous H_2_S synthesis. Cys also contributes to some physiological and pathological processes, such as skeletal muscle wasting and deficiency in the immune system, which lead to health issues including liver damage, lethargy, and a loss of muscle and fat [20]. Moreover, sulfur amino acid metabolism, including methionine (Met) and Cys, plays important catalytic roles in the active sites of many enzymes. For example, Met is primarily metabolized to S-adenosylmethionine, a sulfonium compound that mediates most biochemical methylation reactions [21]. The high nucleophilicity of thiols facilitates the role of Cys as an active site and covalent catalyst, and allows the Cys residue of glutathione to scavenge and detoxify electrophiles during mercapturic acid biosynthesis 5, 6 and peroxide reduction [20]. However, the role and mechanism of these effects are again unclear in mammary glands, particularly in mammary glands with CM.

Therefore, the goal of this study is to systematically identify candidate differentially expressed proteins (DEPs) or target molecules associated with sulfur metabolic processes and endogenous H_2_S in cows with CM using bioinformatics analysis of data-independent acquisition (DIA) proteomics. We also investigate the role and mechanism of the DEPs related to sulfur metabolism and endogenous H_2_S in cows with CM. This research provides support for the prevention and treatment of CM and the development of anti-inflammatory drugs.

## 2. Materials and Methods

### 2.1. Sample Preparation and Collection

Blood samples from lactating Holstein cows with or without CM (n = 6 for each group), as determined by previous veterinary clinical diagnosis (udder examination criteria: redness, hotness, swelling, and painful sensation [1]) were collected from a commercial farm (Wu Zhong City, Ningxia Province, China) and tested for H_2_S concentration. Milk samples (20–25 mL) from the Holstein cows were collected for somatic cell count (SCC) using the method according to the manufacturer’s instructions [22]. The samples were categorized into healthy (SCC ≤ 1 × 10^5^ cells/mL) and CM (SCC ≥ 13 × 10^5^ cells/mL). According to the veterinary clinical diagnosis and SCC results, healthy Holstein cows (categorized as the control group, C) and Holstein cows with CM (categorized as the experimental group, CM) (n = 3 for each group) were selected and transferred to the slaughterhouse of Wuzhong City. Each mammary gland tissue with a volume of 1 cm^3^ was fixed in 4% paraformaldehyde, and the remainder fresh tissues were cut into smaller pieces and immediately stored in liquid nitrogen. This study was approved by the local ethics committee of Gansu Agricultural University, Lanzhou, China.

### 2.2. Bioinformatics Analysis

DIA proteomic sequence data (Table S1) with accession numbers IPX0003382000/ PXD028100 in the ProteomeXchange database (https://www.iprox.cn/page/home.html, accessed on 25 August 2021) were used to identify the DEPs associated with endogenous H_2_S or sulfide synthesis and metabolism in Holstein cows with CM. In this study, we selected significant differences in Gene Ontology (GO) terms and Kyoto Encyclopedia of Genes and Genomes (KEGG) pathways *(p* < 0.05 and *Q* < 0.05) that included four proteins (CBS, CSE/CTH, 3-MST, and CAT) or were related to the sulfur metabolic process as the target proteins to elucidate the functions of endogenous H_2_S. Heat maps, circus graphs, volcano plots, and Upset-Venn diagrams were drawn using R language and the OmicShare online platform [23,24]. Protein and protein interaction networks (PPI) of these DEPs were constructed using STRING v 11.5 [25] and Cytoscape 3.7.1 [26] software including Clue-go and ingenuity pathway analysis (IPA, Ingenuity Systems, http://www.ingenuity.com/, accessed on 25 August 2021) [27].

### 2.3. Endogenous H_2_S Detection

The endogenous H_2_S concentration of the separated serum samples (C and CM groups) was detected using the Micro H_2_S Content Assay Kit (Solarbio, Beijing, China), according to the manufacturer’s instructions. All H_2_S detection was performed using a microplate reader (ReadMax 1900, Shanghai, China) at a wavelength of 665 nm. All experiments were performed at least in triplicate.

### 2.4. Hematoxylin-Eosin (H&E) Staining

Fixed tissues were embedded in paraffin (Solarbio, Beijing, China) and then cut into 5-µm-thick sections using a microtome (Leica, Wetzlar, Germany). The sections were deparaffinized in xylene and dehydrated using an ethyl alcohol gradient. After deparaffinization and rehydration, paraffin-embedded tissue microarray sections were stained with an H&E dye solution set (Servicebio, Wuhan, China). After staining, the sections were dehydrated using increasing concentrations of ethanol and xylene to confirm the histological verification. The sections were then sealed with neutral balsam (Solarbio, Beijing, China). Images were captured using an upright optical microscope and imaging system (Nikon, Tokyo, Japan).

### 2.5. Immunohistochemistry (IHC) Staining and Immunofluorescence (IF) Assay

CTH and CBS proteins were detected using the immunohistochemical standard avidin-biotin-peroxidase complex (ABC) staining system (Bioss, Beijing, China) [24,28]. Antigen retrieval was performed by heating the samples in a microwave oven (750 W for 10 min) and cooling them to room temperature. Endogenous catalase deactivation was performed by immersing slides in 0.3% (*v*/*v*) hydrogen peroxide (H_2_O_2_) at room temperature. IHC staining was performed using a Histostain-SP (Streptavidin-Peroxidase) kit (Bioss, Beijing, China) according to the manufacturer’s instructions [24,28]. The sections were incubated with rabbit anti-CBS (1:500, Abcam, Cambridge, UK) and mouse monoclonal anti-CTH (1:150, Abcam, Cambridge, UK) overnight at 4 °C in a wet box. Finally, positive signals were visualized using a 3-diaminobenzidine kit (Solarbio, Beijing, China). For tissular IF staining, the paraffin sections were incubated with 3% H_2_O_2_ and blocked with Normal Donkey Serum (1:20, Solarbio, Beijing, China) at room temperature; the antigen retrieved sections were used for IF staining and labeled with different colors of immunoglobin G, as described previously [29]. The primary antibodies are rabbit anti-CBS (1:400, Abcam, Cambridge, UK), mouse monoclonal anti-CTH (1:150, Abcam, Cambridge, UK), and anti-cytokeratin 18 (CK-18, 1:100, Bioss, Beijing, China) were incubated at 4 °C overnight. The nuclei were localized using 4′, 6-diamidino-2-phenylindole (DAPI, Solarbio, Beijing, China). All immunostaining assays were performed at least in triplicate. Images were captured using a fluorescence microscope (Olympus Corporation, Tokyo, Japan). The gray value of positive expression products in the IHC sections was quantified and scanned using Image-Pro Plus 6.0 (Media Cybernetics Co., Rockville, MD, USA). Group C was used as the control. At least two visual fields were randomly selected from each slice, and all optical density scans were repeated at least in triplicate.

### 2.6. RNA Isolation, cDNA Synthesis, and qRT-PCR Assays

Total RNA was extracted from the mammary glands using a FastPure RNA isolation kit (Vazyme, Nanjing, China) following the manufacturer’s instructions, then used for cDNA synthesis. RNA was quantified using a NanoDrop-8000 (Thermo Fisher Scientific, Waltham, MA, USA), and RNA integrity was assessed by 1% denaturing formaldehyde agarose gel electrophoresis (Biowest Regular Agarose, Castropol, Spain). One microgram of total RNA was subjected to reverse transcription to cDNA using the Evo M-MLV RT Kit (Agbio, Hunan, China), and quantitative reverse transcription-polymerase chain reaction (qRT-PCR) was performed, as described previously [24]. The relative expression levels of *CTH* and *CBS* mRNA in the mammary gland tissues were detected using qRT-PCR, which was performed with 2×SYBR^®^ Green *pro Taq* HS Premix (Agbio, Hunan, China) following the manufacturer’s instructions and diluted to 80 ng/µL cDNA templates using a standard two-step reaction. *Glyceraldehyde-3-phosphate dehydrogenase (GAPDH)* was used as an endogenous control. qRT-PCR primers (Table S2) were designed using Premier software (version 5.0) [24,28] and synthesized by Qinke Biotech Co., Ltd. (Yangling City, Shanxi Province, China). qRT-PCR was performed using the Light Cycler 96 real-time system (Roche, Switzerland). All qRT-PCR assays were performed at least in triplicate. The procedures and calculations were performed as previously described [24,30].

### 2.7. Western Blot

The relative expression of CTH and CBS proteins in the mammary glands of the C and CM groups was examined by western blotting. Total proteins were isolated from mammary tissues using a cold RIPA (Radioimmunoprecipitation assay) buffer (Solarbio, Beijing, China) supplemented with 1 mM phenylmethylsulfonyl fluoride (PMSF, Solarbio, Beijing, China) and a protease inhibitor mixture (PIC, Solarbio, Beijing, China). The protein concentration was assessed using a BCA (Bicinchoninic Acid) protein assay kit (BOSTER, Wuhan, China) according to the manufacturer’s instructions. Total protein samples (30 µg) were electrophoresed on a sodium dodecyl sulfate-polyacrylamide gel (SDS-PAGE). The blots were transferred onto a PVDF (polyvinylidene fluoride) membrane (Millipore CAT, Billerica, MA, USA) and blocked with Tris-HCl buffer (Solarbio) containing 5% (*w*/*v*) non-fat powdered milk (Solarbio, Beijing, China) at room temperature. The primary antibodies are rabbit anti-CBS (1:3000, Abcam, Cambridge, UK), mouse monoclonal anti-CTH (1:1000, Abcam, Cambridge, UK), and anti-β-actin (1:4000, Bioss, Beijing, China) were incubated at 4 °C overnight. Subsequent procedures were performed as previously described [28,30]. The optical densities of the bands were quantified and scanned using Image-Pro Plus 6.0 (Media Cybernetics Co., Rockville, MD, USA). β-actin was used as a control. All immunoblot assays were performed at least in triplicate.

### 2.8. Statistical Analysis

All data are presented as the mean ± SEM, unless otherwise indicated. Statistical analysis was performed using SPSS version 22.0 (SPSS Inc., Chicago, IL, USA). H_2_S concentration, IHC, qRT-PCR, and western blot data were analyzed by Student’s *t*-test (between two groups) or one-way ANOVA analysis (within multiple groups). Graphs were constructed using GraphPad Prism 9.0 (GraphPad Software Inc., San Diego, CA, USA). Statistical significance was set to *p* < 0.05.

## 3. Results

### 3.1. Identification of the Candidate DEPs Related to Sulfur Metabolism from the GO Terms

**Figure 1:**
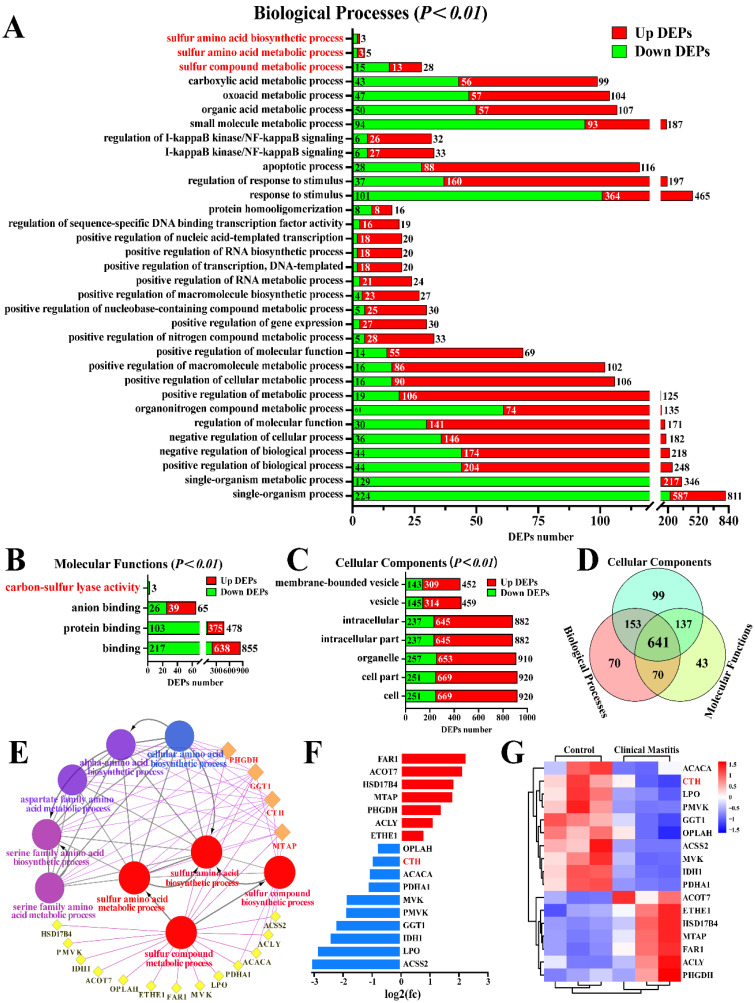
Identification of candidate DEPs and GO terms related to sulfur metabolism. (**A**–**C**) Candidate DEPs and GO terms including biological process (**A**), molecular function (**B**) and cellular component(**C**) related to sulfur metabolism; x-axis represents the number of DEPs. y-axis represents the GO terms. (**D**) Venn diagram of candidate DEPs in the BP, MF, and CC groups. (**E**) PPI network analysis of 17 DEPs and nine GO terms related to sulfur metabolism. (**F**) Relative expression levels of 17 DEPs quantified by DIA proteomics; x-axis represents the log2(FC) values. (**G**) Heat map of 17 DEPs related to sulfur metabolism.

The candidate DEPs related to sulfur metabolism were screened from the biological processes (BP), molecular functions (MF), and cellular components (CC) of GO terms according to 3739 DEPs (Figure 1). A total of 33 BP, four MF, and seven CC were identified from the significantly differentially expressed GO terms (*p* < 0.05 and *Q* < 0.05), particularly the sulfur compound metabolic process, sulfur amino acid biosynthetic process, sulfur amino acid metabolic process, and carbon-sulfur lyase activity (Figure 1A–C). After overlapping the repeated DEPs, 1205 DEPs (Table S3), including 934 DEPs in BP, 891 DEPs in MF, and 1030 DEPs in CC, were identified from these GO terms. A total of 641 co-expressed DEPs were identified, of which CTH was the only DEP among the four demonstrated proteins (Figure 1D). We then constructed the interaction network of these GO terms and the DEPs, and the results suggested that nine GO terms, including 17 DEPs, directly interacted with, and played a crucial role in sulfur or H_2_S metabolism (Figure 1E). Compared to the relative expression levels of these 17 DEPs in the C group, seven DEPs were upregulated, and 10 DEPs were downregulated in the CM group (Figure 1F). A heat map of these 17 DEPs showed that they were differentially expressed in the mammary gland tissues of the C and CM groups (Figure 1G). These results revealed that the expressed variations in DEPs (especially CTH) related to sulfur metabolism or endogenous H_2_S are closely related to CM in Holstein cows.

### 3.2. Identification of Candidate DEPs Related to Sulfur Metabolism from KEGG Pathways

**Figure 2:**
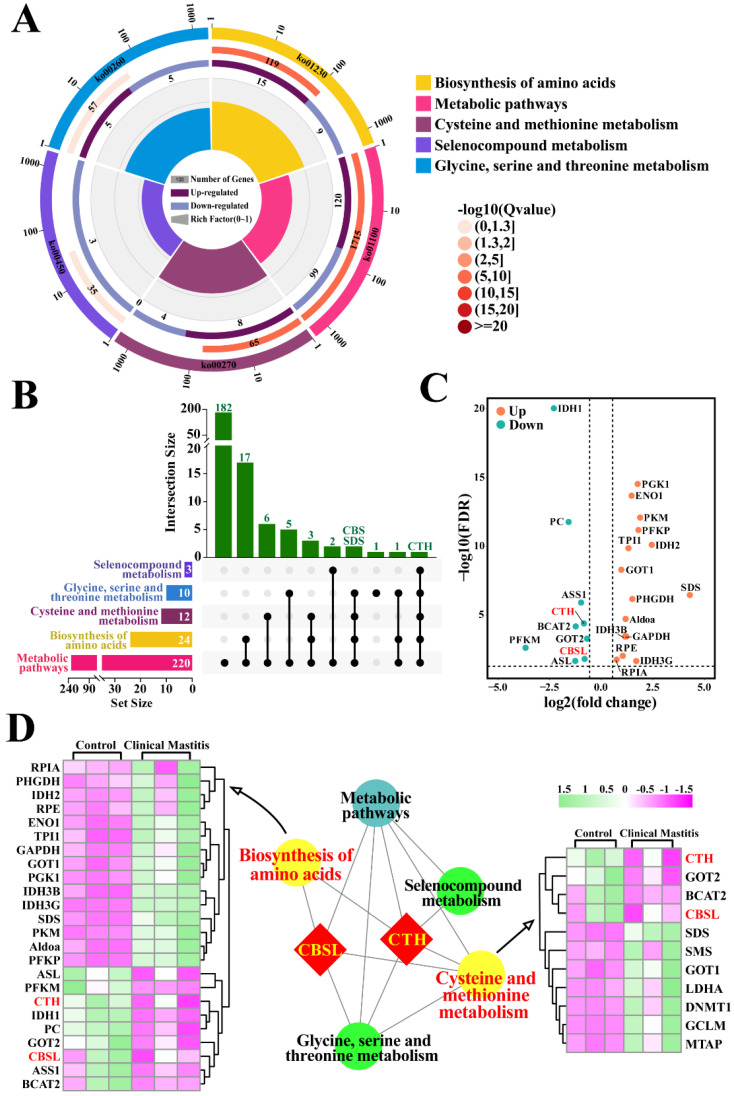
Identification of candidate DEPs and KEGG pathways related to sulfur metabolism. (**A**) Enrichment circle diagram of KEGG pathways related to sulfur metabolism. Color of the outermost layer represents the different KEGG classifications related to sulfur metabolism. Second layer contains information on the number of DEPs and the degree of significant enrichment. Third layer shows the upregulation and downregulation of DEPs. Fourth layer represents the enrichment factor for each pathway. (**B**) Upset-Venn diagram of five KEGG classifications related to sulfur metabolism. (**C**) Volcano plots of the 24 DEPs, including eight downregulated and 16 upregulated DEPs. (**D**) PPI network and heat map analysis of amino acid biosynthesis and cysteine and methionine metabolism.

The candidate DEPs related to sulfur metabolism were also selected from the KEGG pathways with *p* < 0.05 and *Q* < 0.05, according to 3739 DEPs and 67 KEGG pathways (Figure 2). Five significantly different pathways were selected, including 220 DEPs (Table S4), particularly those related to the biosynthesis of amino acids (24 DEPs) and cysteine and methionine metabolism (12 DEPs) (Figure 2A). We then constructed the Upset-Venn diagram of these five pathways. The results suggested that CTH was the only DEP shared among these five pathways, whereas CBS and L-serine dehydratase/L-threonine deaminase (SDS) were co-expressed in four pathways (Figure 2B). Vo-lcano plots were then constructed, taking the amino acid biosynthesis pathway as an example. A total of 24 DEPs were screened, including eight downregulated and 16 upregulated DEPs, which accounted for 2.65% of these pathways (Figure 2C). Notably, downregulated DEPs of CBS, one of the four demonstrated proteins, were identified in four pathways. The interaction network of these pathways was also constructed, which suggested that CTH participated in all five pathways, whereas CBS was involved in four pathways, particularly the biosynthesis of amino acids and the cysteine and methionine metabolism pathways. The DEPs included in these two pathways were significantly differentially expressed in the mammary glands of the C and CM groups (Figure 2D). These results further indicate that CTH and CBS control the relationship between sulfur metabolism or endogenous H_2_S and CM in Holstein cows.

### 3.3. Location Analysis and Measurement of CBS and CTH Proteins and Endogenous H_2_S

**Figure 3:**
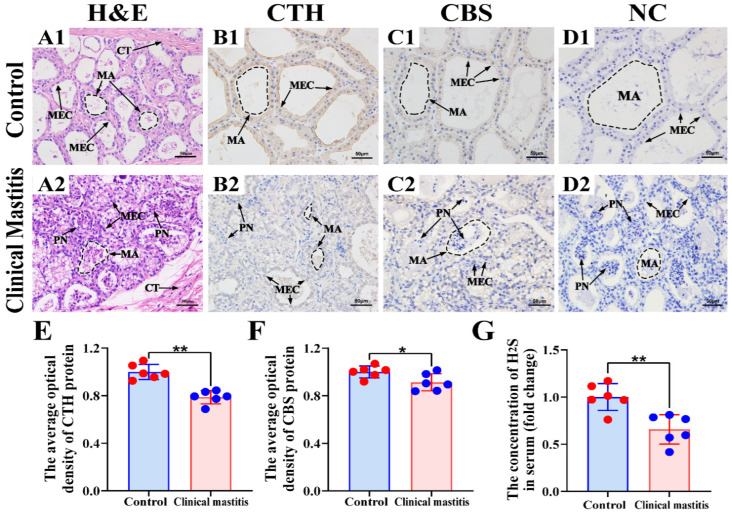
Location analysis of CBS and CTH proteins and measurement of endogenous H_2_S in the mammary glands. (**A**) Pathological variation of the mammary glands of the C (**A1**) and CM (**A2**) groups (400×). (**B**,**C**) Intracellular location analysis of CBS and CTH proteins in the mammary glands of the C (**B1,C1**) and CM (**B2,C2**) groups (400×). (**D**) Negative control of the mammary glands of the C (**D1**) and CM (**D2**) groups. (**E**,**F**) The gray value of positive expression of CTH and CBS proteins about IHC sections were scanned and quantified. (**G**) Endogenous H_2_S concentrations in the serum of the C and CM groups. CT, connective tissue. MA, mammary alveoli. MEC, mammary epithelial cells. PN, phagocytic neutrophils. C represents control, CM represents clinical mastitis. H_2_S concentration and IHC data were analyzed by Student’s *t*-test (between two groups) or one-way ANOVA analysis (within multiple groups), and expressed as mean ± SEM. * represents *p* < 0.05 and ** represents *p* < 0.01.

In this study, we focused on mammary alveoli (MA) and mammary epithelial cells (MECs) in the mammary glands of the C and CM groups (Figure 3). H&E staining showed that, in the C group, the MA exhibited large alveolar luminal areas without evidence of inflammation, and MECs were intact and arranged neatly in the mammary glands (Figure 3A1). Conversely, in the CM group, alveoli had collapsed and infiltrated a large number of inflammatory cells, and the MECs were exfoliated in the mammary glands (Figure 3A2). IHC results suggested that positive staining of CTH and CBS was distributed mainly in the cytoplasm of MECs with differential staining degrees (Figure 3B1,B2,C1,C2). The negative control group had no positive staining for CTH and CBS proteins (Figure 3D1,D2). Positive staining of CTH and CBS proteins in the C group was stronger than that in the CM group (Figure 3E,F). Moreover, the degree of positive staining of the CTH protein was also substantially stronger than that of the CBS protein in both C and CM groups. Endogenous H_2_S concentration in the serum of C and CM groups showed that endogenous H_2_S production was significantly lower in the CM group than in the C group (Figure 3G). This suggests that CTH may play an important regulatory role in cow mastitis.

### 3.4. Co-Location Analysis of CBS and CTH Proteins in Mammary Glands

**Figure 4:**
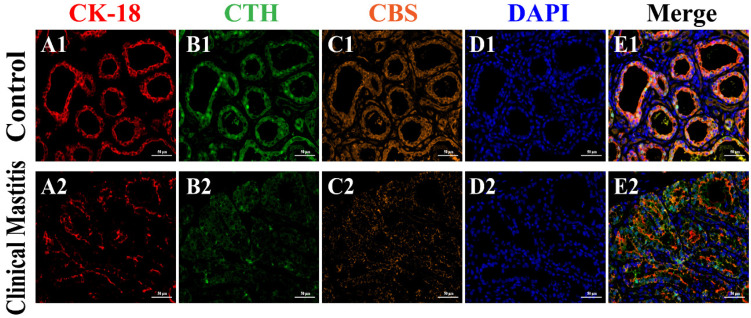
Co-location analysis of CBS, CTH, and CK-18 proteins in the mammary glands (400×). (**A**–**C**) Cellular localization of CK-18 (red), CTH (green), and CBS (orange) proteins in the mammary glands of the C (**A1**,**B1**,**C1**) and CM (**A2**,**B2**,**C2**) groups, respectively (400×). (**D**) Nuclei stained with DAPI (blue) in the mammary glands of the C (**D1**) and CM (**D2**) groups (400×). (**E**) Merged colocalization of CK-18, CTH, and CBS proteins in the mammary glands of the C (**E1**) and CM (**E2**) groups (400×). C, represents control. CM, represents clinical mastitis.

IF signals of CBS, CTH, and CK-18 proteins were clearly present in the mammary glands of both C and CM groups (Figure 4). Additionally, CK-18, a specific marker of epithelial cells, appeared in the cytoplasm of MECs (Figure 4A1,A2). According to the positive signals of CK-18, the histological structures of the MA were intact in the mammary glands of the C group but incomplete in the mammary glands of the CM group. IF signals of CTH and CBS proteins were also present in the mammary glands of the C and CM groups, particularly in MECs (Figure 4B1,B2,C1,C2). According to the positive IF signals, the CTH and CBS proteins in the C group were stronger than those in the CM group. Co-location analysis revealed that CK-18, CTH, and CBS were co-localized in the cytoplasm of MECs. The positive IF signals of CTH and CBS were also stronger in the C group than in the CM group, which agrees with the results of IHC staining (Figure 3E1,E2). These results suggest that the functions of CTH and CBS proteins are closely related to epithelial cells.

### 3.5. Expression Patterns of CTH and CBS mRNA and Protein in Mammary Glands

**Figure 5:**
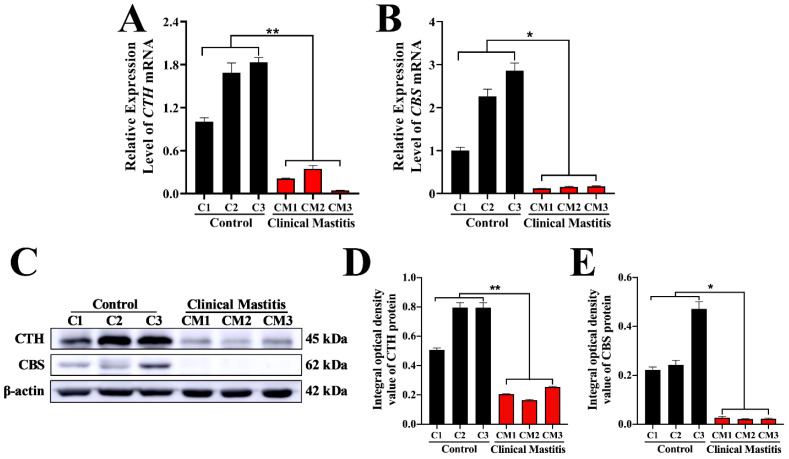
Relative expression levels of *CBS* and *CTH* mRNA and proteins in the mammary glands. (**A**,**B**) Expression levels of *CBS* and *CTH* mRNA in the mammary glands of the C and CM groups. *GAPDH* mRNA was used as the internal control. (**C**) Western blot analysis of CBS, CTH, and β-actin proteins in the mammary glands of the C and CM groups. (**D**,**E**) Relative integral optical density of CBS and CTH in the mammary glands of the C and CM groups. C, represents control. CM, represents clinical mastitis. The data were analyzed by Student’s *t*-test (between two groups) or one-way ANOVA analysis (within multiple groups), and expressed as mean ± SEM. * represents *p* < 0.05 and ** represents *p* < 0.01.

According to the qRT-PCR results, the relative expression levels of *CTH* and *CBS* mRNA in the CM groups were significantly downregulated compared to those in the C groups (Figure 5A,B). Western blotting results showed that the CTH protein was detected in both C and CM groups, whereas the CBS protein was only detected in the C group (Figure 3C). Statistical results of western blotting indicated that the relative expression levels of CTH and CBS proteins in the CM group were also significantly downregulated compared to those in the C group (Figure 3D,E). Generally, these results indicate that the expression levels and distribution patterns of CTH and CBS in the C group were significantly higher than those in the CM group. In particular, it should be noted that the difference in CTH between group C and CM is more significant than that of CBS.

## 4. Discussion

As an alternative to current CM prevention and treatment methods employed in the dairy industry, antibiotic substitutes that do not generate antibiotic resistance or side effects are a research priority. A lack of such drugs and the target molecules of CM has led to the common occurrence of CM in large-scale farming. Endogenous H_2_S, which is a gaseous transmitter synthesized by CBS, CSE/CTH, 3-MST, and CAT, has been implicated in many physiological and pathological processes. Studies have shown that H_2_S has anti-inflammatory, anti-tumor, regulating ion channels, protecting cardiovascular and antioxidant effects, particularly in inflammatory responses and adaptive immunity [10,11]. However, it is unclear whether H_2_S could be used as a target for the prevention and treatment of CM. Moreover, the role and synthesis of endogenous H_2_S enzymes in dairy cows remain ambiguous.

The identification of protein repertoires in the milk, serum, and mammary glands of cows suffering from CM is ideal for identifying potential biomarkers for the development of rapid diagnostic tests [27,31,32]. This approach has been used to screen markers, such as Toll-like receptors, cathelicidins, cathepsins, and apoptotic factors [27,33]. Using healthy Holstein cows as controls, we screened DEPs in the mammary glands of Holstein cows with CM using DIA proteomic data, focusing on the DEPs related to endogenous H_2_S synthesis and sulfur metabolism. Four direct GO terms and 40 indirect GO terms, including 641 DEPs, were selected according to significant GO terms, which indicates that these DEPs were related to sulfur metabolism and H_2_S synthesis. Sulfur amino acids, including Met and Cys, are two important amino acids involved in animal metabolism and important sources of sulfur or sulfhydryl. These are also the substrates used for H_2_S synthesis. H_2_S can be generated by a number of mechanisms from l-homocysteine and Cys via the Met transsulfuration pathway or from dietary Cys [34]. Moreover, sulfur amino acids are abundant in milk, particularly in the mammary glands, which also provide substrates for H_2_S synthesis. These findings indicate that endogenous H_2_S plays an important role in mammary glands. Among the DEPs, 17 were involved in multiple GO terms and three DEPs (CTH, CBS, and SDS) were involved in five KEGG pathways related to the biosynthesis of amino acid metabolism, especially that of sulfur amino acids. At present, it is known that CBS mainly condenses L-homocysteine with L-Cys (β-replacement) and desulfurizes L-Cys to L-serine (β- elimination), and CTH desulfurizes L-Cys to pyruvate (α, β-elimination) to synthesize H_2_S. This is consistent with the reactions found in Cysteine and methionine metabolism, and Glycine, serine, and threonine metabolism, based on the five KEGG pathways. Previous studies have shown that the majority of endogenous H_2_S is produced by CBS and CTH, using L-Cys or homocysteine as substrates [34,35]. These two enzymes are pyridoxal-50-phosphate-dependent and responsible for Cys metabolism [18,19,20]. There are at least two mechanisms for the release of H_2_S. One is that H_2_S produced by enzyme catalysis exists in a free state and directly plays the role of signal molecules in the body. Another possible mechanism is that the generated H_2_S is stored in cells in the form of bound sulfane sulfur to be released in response to physiological signals. The specific existing form of H_2_S is related to the pH value of its environment. Since the reducing activity of mercaptan is higher in alkaline conditions than at a neutral pH, H_2_S release is observed when the pH is higher than 8.4 [18]. Previous studies have also shown that ATP-citrate synthase isoform (ACLY) [36], D-3-phosphoglycerate dehydrogenase (PHGDH) [37], gamma-glutamyltranspeptidase 1 (GGT1) [38], S-methyl-5′-thioadenosine phosphorylase (MTAP) [21], persulfide dioxygenase 1 (ETHE1) [39], and SDS [40] can mediate H_2_S synthesis via different pathways, such as fatty acid and glutathione metabolism, sulfur compound metabolic processes, and cysteine and methionine metabolism. In addition, some DEPs, such as phosphomevalonate kinase (PMVK), mevalonate kinase (MVK), and pyruvate dehydrogenase E1 component subunit alpha (PDHA1), lack relevant evidence for their participation in sulfur metabolism. These DEPs play important roles in cell apoptosis, proliferation, and inflammation [41,42]; thus, the functions of these DEPs in cows with CM require further study. IHC and IF staining results indicated that MECs could produce H_2_S via DEPs related to sulfur amino acid metabolism, particularly CTH. Moreover, the H_2_S produced in MECs may be associated with MEC functions. MECs can synthesize and secrete inflammatory chemokines and pro-inflammatory cytokines such as interleukin-1 beta (IL-1β), interleukin-6 (IL-6), interleukin-8 (IL-8), and tumor necrosis factor (TNF-α) [43], which are crucial for the occurrence of mastitis. MECs, an important part of innate immunity, have been widely studied for their role in the prevention and treatment of mastitis. Previous studies have shown that deficiency of the *CTH* gene increases the sensitivity of mice to LPS-induced inflammation, as evidenced by the higher expression and secretion of interleukin-2 (IL-2), interleukin-4 (IL-4), IL-6, and TNF-α in CTH-KO mice compared to wild-type mice [44]. H_2_S may also exert bidirectional effects on mammary gland ductal development, promoting ductal development at low concentrations but inhibiting it at high concentrations [45,46]. As well-known inflammatory cytokines or chemokines, *TNF-α*, *IL-1β*, *IL-6*, and *IL-8* are involved in various types of inflammatory responses and are crucial in inflammatory processes during bacterial infection in bovine mammary glands [47]. Our findings suggest that CTH/H_2_S is related to mammary gland inflammation. The results of qRT-PCR, western blotting, and endogenous H_2_S concentration showed that *CBS* and *CTH* mRNA and protein levels in the CM group were significantly downregulated compared to those in the C group. Therefore, reduced endogenous H_2_S and downregulated expression of H_2_S-produced proteins could be one of the reasons for the occurrence and development of CM in dairy cows. It has been suggested that biphasic effects of the CTH/H_2_S system are anti-inflammatory at low concentrations and pro-inflammatory at high concentrations (200–500 mM or greater) [10,11,48]. Therefore, the anti-inflammatory effect of H_2_S at physiological concentration can provide the possibility for the prevention and treatment of CM in dairy cows. At the same time, these findings can not only enrich people’s understanding of the relationship between H_2_S and inflammation, but also provide a new target for the development of anti-inflammatory drugs based on H_2_S exogenous donors. However, the specific mechanism of the anti-inflammatory effect of H_2_S and its donors needs to be further studied.

In conclusion, endogenous H_2_S has a regulatory effect on CM, and CTH/H_2_S is correlated with the occurrence and development of CM in Holstein cows. These findings are expected to promote research related to the molecular mechanism of the CTH/H_2_S system and provide support for the prevention and treatment of CM and the development of anti-inflammatory drugs.

## 5. Conclusions

In this study, 17 DEPs related to sulfur compound metabolism and sulfur amino acid metabolism were obtained from the DIA proteomics sequence database. Among these DEPs, CTH and CBS exhibited important roles in dairy cows with CM. Compared to the C group, the endogenous H_2_S concentration in the serum of the CM group was significantly decreased. CTH and CBS proteins were present mainly in the cytoplasm of MECs, and the relative expression levels of *CTH* and *CBS* mRNA and protein in the CM group were significantly downregulated compared to those in the C group. These results suggest that CTH/H_2_S is correlated with CM in Holstein cows and that endogenous H_2_S has a regulatory role in Holstein cows with CM.

## Data Availability

The data that support the findings of this study are available from the corresponding author upon reasonable request.

## Supplementary Materials

The following supporting information can be downloaded at: https://www.mdpi.com/article/10.3390/ani12111451/s1. Table S1: The 3739 DEPs generated from the DIA proteomics sequence database. Table S2: qRT-PCR primer sequences. Table S3: The 1205 candidate DEPs related to sulfur metabolism identified from 988 GO terms. Table S4: The 220 candidate DEPs related to sulfur metabolism identified from 67 pathways.
