# Supplementary material for: Sulfur Amino Acid Metabolism and the Role of Endogenous Cystathionine-γ-lyase/H2S in Holstein Cows with Clinical Mastitis
Source: Animals (Basel). 2022 Jun 4;12(11):1451. doi: 10.3390/ani12111451 (PMC9179249; doi:10.3390/ani12111451)
Supplement: Supplementary file 1 [file animals-12-01451-s001.zip › Supplementary Materials Raw data description.pdf]

Supplementary Materials:

Table S1: The 3,739 DEPs generated from the DIA proteomics sequence database.

Table S2: qRT-qPCR primer sequences.

Table S3: The 1,205 candidate DEPs related to sulfur metabolism identified from 988 GO terms.

Table S4: The 220 candidate DEPs related to sulfur metabolism identified from 67 pathways.
