# Supplementary material for: Sulfur Amino Acid Metabolism and the Role of Endogenous Cystathionine-γ-lyase/H2S in Holstein Cows with Clinical Mastitis
Source: Animals (Basel). 2022 Jun 4;12(11):1451. doi: 10.3390/ani12111451 (PMC9179249; doi:10.3390/ani12111451)
Supplement: Supplementary file 1 [file animals-12-01451-s001.zip › Table S2 (RT-qPCR primer sequences).pdf]

Table S2. RT-qPCR primer sequences.

Primer name	Accession NO.	Sequence 5'-3'	Product Length
GAPDH	NM_001034034.2	F: GGTCACCAGGGCTGCTTT<br>R: CTGTGCCGTTGAACTTGC	128 bp
CBS	NM_001102000.2	F: CCGGAGAAGATGAGCACAGA<br>R: ATGTCATAGTGAGCGAGGGG	197 bp
CTH	NM_001024567.1	F: AGGGCTCTCTTCAACATGCT<br>R: AAGCCCACTGAGAGTCGAAT	184 bp
IL-1β	NM_174093.1	F: TCAATAAAGTGCAAACTCCAGGACA<br>R: CTTGCACAAAGCTCATGCAGAA	133 bp
IL-6	NM_173923.2	F: CCTTCACTCCATTCGCTGTCT<br>R: TCCTGATTTCCCTCATACTCG	391 bp
IL-8	NM_173925.2	F: GCTGGCTGTTGCTCTCTTG<br>R: GGGTGGAAAGGTGTGGAATG	126 bp
TNF-α	NM_173966.3	F: AAGCCTCAAGTAACAAGCCGGTAG<br>R: TCACACCGTTGGCCATGAG	108 bp
TLR4	NM_174198.6	F: CATCATCTTCATCGTCCTG<br>R: ATCTGCTGTTCCTTCTGG	190 bp
